# Impact of Vitamin D on Chronic Kidney Diseases in Non-Dialysis Patients: A Meta-Analysis of Randomized Controlled Trials

**DOI:** 10.1371/journal.pone.0061387

**Published:** 2013-04-23

**Authors:** Lijuan Xu, Xuesi Wan, Zhimin Huang, Fangfang Zeng, Guohong Wei, Donghong Fang, Wanping Deng, Yanbing Li

**Affiliations:** 1 Department of Endocrinology, The First Affiliated Hospital of Sun Yat-sen University, Guangzhou, Guangdong Province, China; 2 Guangdong Provincial Key Laboratory of Food, Nutrition and Health, School of Public Health, Sun Yat-sen University, Guangzhou, Guangdong Province, China; University of Milan, Italy

## Abstract

**Background and Objectives:**

Recent studies have supported a role for both newer and more established vitamin D compounds in improving proteinuria, although systematic evaluation is lacking. Furthermore, concerns remain regarding the influence of vitamin D on the progression of renal function. We analyzed the efficacy and safety of vitamin D in non-dialysis patients and compared the use of newer versus established vitamin D compounds by performing a meta-analysis of randomized controlled trials.

**Design:**

A literature search of PubMed (1975 to September, 2012), EMBASE.com (1966 to September, 2012) and Ovid EBM Reviews (through September, 2012) was conducted.

**Results:**

Eighteen studies were eligible for final inclusion; of these, six explored the effects of vitamin D on proteinuria, twelve studied the effects of supplementation on renal function, and fifteen discussed the incidence of hypercalcemia. Compared to the placebo or no interference, both the newer and established vitamin D sterols reduced proteinuria to a similar extent (RR, 2.00; 95% CI, 1.42 to 2.81). No decrease in the glomerular filter rate was observed (SMD, −0.10; 95%CI, −0.24 to 0.03), and the risk for dialysis initiation was 1.48 (95% CI, 0.54 to 4.03) with vitamin D treatment. Additionally, there was an increased risk of hypercalcemia for patients treated with either newer or established vitamin D compounds as compared with the controls (RR, 4.78; 95% CI, 2.20 to 10.37). The head-to-head studies showed no differences in the effects of either newer or established compounds on proteinuria or the risk of hypercalcemia. No serious adverse events were associated with the administration of vitamin D.

**Conclusions:**

Vitamin D therapy appears to decrease proteinuria and have no negative influence on renal function in non-dialysis patients. But the occurrence of hypercalcemia should be evaluated when vitamin D is provided. No superiority for newer versus established vitamin D analogue is found.

## Introduction

End-stage renal disease (ESRD) imposes significant health and economic burdens on both individuals and communities [1]. Microalbuminuria is one of the earliest clinical manifestations of nephropathy and is associated with substantial risk for progressive kidney disease. Additionally, albuminuria predicts cardiovascular events, all-cause mortality and hospitalization for congestive heart failure [2]. Recent data have shown that increased proteinuria and decreased glomerular filtration rate (GFR) serve as independent predictors of all-cause mortality [3], [4]. Thus, reducing proteinuria and protecting kidney function at the disease stages prior to dialysis are pivotal for preventing long-term kidney loss and other adverse events.

Renin-angiotensin system (RAS) inhibitors can reduce proteinuria and delay kidney dysfunction in patients with chronic kidney disease (CKD), but are unsuitable for those with advanced renal dysfunction due to the potential for renal deterioration and hyperkalemia. The exploration of other therapeutic modalities is urgently needed for CKD treatment. Although animal experiments have revealed that vitamin D can reduce proteinuria [5], the majority of existing clinical data have focused on the effect of vitamin D on mineral metabolism and bone diseases related to secondary hyperparathyroidism. Of the limited clinical studies that have explored extra-skeletal benefits of vitamin D, the VITAL trial (selective vitamin D receptor activation with paricalcitol for the reduction of albuminuria), a well-designed and relatively large-scale study, has shown promising but borderline significant results concerning albuminuria improvement [6]. In addition, it remains unclear whether vitamin D treatment may harm renal function. Vitamin D therapy has been widely used in the management of CKD, traditionally in the form of ergocalciferol (vitamin D2), cholecalciferol (vitamin D3), calcitriol (1, 25 dihydroxyvitamin D3) and alfacalcidol (1α- hydroxyvitamin D3). However, the newer vitamin D analogues, including paricalcitol, doxercalciferol, 22-oxacalcitriol and falecalcitriol, play an increasingly important role in CKD treatment based on the experimental results of similar or better suppression of parathyroid hormone and possibly less calcemic effect compared with established vitamin D sterols [7]. While it is still uncertain whether newer compounds are superior to the established ones in terms of albuminuria improvement, renal function protection, hypercalcemia and other side effects reduction. The different forms of vitamin D compounds were listed in Table 1.

**Table 1:** Vitamin D and derivatives.

Vitamin D2 and derivatives		Vitamin D3 and derivatives
**The established vitamin D compounds**		
Parent compound	Vitamin D2	Vitamin D3
synonym	Ergocalciferol	Cholecalciferol
Product of first hydroxylation	25-hydroxyvitamin D2	25-hydroxyvitamin D3
synonym	Ercalcidiol	Calcidiol
Product of second hydroxylation	1,25-Dihydroxyvitamin D2	1,25-Dihydroxyvitamin D3
synonym	Ercalcitriol	Calcitriol
**The newer vitamin D analogues**		
Full term	1alpha-hydroxyergocalciferol	22-oxacalcitriol
synonym	doxercalciferol	Maxacalcitol
Full term	19-Nor-1,25-Dihydroxyvitamin D2	F6-1α,25-Dihydroxyvitamin D3
synonym	Paricalcitol *	falecalcitriol

* In some literatures, paricalcitol is considered as the derivative of calcitriol.

Given the fact that vitamin D is generally deficient and metabolically disordered in patients with CKD [8], [9], supplementation of vitamin D may be significant throughout CKD evolution, especially at early and moderate stages. To our knowledge, few comprehensive meta-analyses and systematic reviews have explored the influence of vitamin D on proteinuria and the progression of CKD in non-dialysis patients or compared treatments between newer and more established sterols. In this regard, we performed a meta-analysis to clarify these issues, and we also evaluated hypercalcemia and other adverse events. The protocol of this analysis is available in File S1 and the search strategies are listed in File S2.

## Design and Methods

### Study inclusion and exclusion criteria

Data from randomized controlled clinical trials (RCTs) that included patients receiving vitamin D in the study group and patients receiving placebo or no medications as controls were eligible for analysis. RCTs that compared newer and established vitamin D analogues were also included. Subjects who suffered from CKD should have no need for dialysis or renal transplantation at baseline. We considered the parameters of albuminuria, GFR, the risk of hypercalcemia and other adverse effects in these trials. The exclusion criteria consisted of incomplete relevant parameters required for our analysis, as unobtainable from the respective author and unable to be analyzed by statistical methods. No restriction was set for language, publishing year or country to maximize the extent of the searches.

### Data search strategies

We performed literature searches of PubMed (1975 to September, 2012), EMBASE.com (1966 to September, 2012) and OvidSP (through September, 2012) for the key words “vitamin d” or “vitamin d2” or “vitamin d3” or “calciferol” or “calcitriol” and “kidney disease” or “nephropathy” with the limitation of “controlled clinical trial”. Detailed data search strategies are given in File S2. Google Scholar was searched as a complementary measure for full-text articles. The EMBASE.com database is composed of Embase (from 1974) and majority of data from Medline (from 1966). OvidSP contains seven sub-databases including the Cochrane Library. Abstracts presented at meetings of the American Society of Nephrology, National Kidney Foundation, World Congress of Nephrology, American Diabetes Association, European Association for the Study of Diabetes and International Diabetes Federation in recent years were searched for additional studies. We used the Endnote X4 program for literature management and selection.

### Data extracted

The following information was summarized by using a predefined data collection form: title, the first author's name, country, mean age, year of publication, drug dosage, controls and causes of CKD. For binary outcomes, the number of cases and controls was recorded. For continuous data, the numbers, mean values and standard deviations of changes from baseline in the study group and the control group were recorded. If the 95% confidence interval was provided instead of the standard deviation, the standard deviation was calculated based on the equation provided in Cochrane Handbook. If the baseline and final standard deviations were given and the changes in the standard deviations were unknown, the correlation coefficient method advised by Follmann was used to calculate the values [10].

Two reviewers (Dr. LJX and Dr. XSW) screened the search results based on the inclusion and exclusion criteria. The two reviewers independently extracted useful data from the selected trials. When it was considered desirable and potentially useful, we contacted the investigators for additional information. Discrepancies between the two reviewers were arbitrated by Professor YBL. Relevant missing data were sought by contacting the original author of the respective study.

The following parameters were accumulated: 1) albuminuria improvement (the numbers of patients who had a proteinuria reduction after treatment were recorded, according to the urine albumin/creatine ratio or 24-hour urine protein excretion); 2) GFR changes (GFR was calculated according to the Modification of Diet in Renal Disease (MDRD) equation, the Cockcroft-Gault method, estimations of the continuous infusion of iothalamate, or predictions of the creatinine clearance rate (CCR) in the original trials, and differences in GFR changes were compared between the study and control groups); 3) incidence of hypercalcemia (hypercalcemia was defined as concentrations of serum calcium above 2.54∼2.80 mmol/L (10.2∼11.2 mg/dL)); and 4) adverse events (all adverse events except for hypercalcemia were summarized in our analysis).

### Study quality assessment

We used Revman 5.1 software (the Cochrane Collaboration, Copenhagen, Denmark) to evaluate the study quality. Two reviewers (Dr. LJX and Dr. FFZ) conducted these assessments independently, and disagreements were resolved through discussion between the two reviewers. The evaluation criteria consisted of the following: 1) random sequence generation; 2) allocation concealment; 3) blinding of participants and personnel; 4) blinding of outcome assessment; 5) incomplete outcome data; 6) selective reporting; and 7) other bias.

### Statistical analysis

Standardized mean differences (SMD) and 95% confidence intervals (CI) were presented to compare the measurement data changes. SMD was used as a summary statistic in our analysis because the data for GFR conformed to the normal distribution; however, the measurement methods varied, and it was necessary to standardize the results to a uniform scale before they could be combined. Dichotomous data were expressed as risk ratios (RR) and 95% CI. In our analysis, the numbers of patients with proteinuria reduction, renal deterioration, hypercalcemia and other events were considered dichotomous data. Heterogeneity was analyzed using a χ-squared test on n-1 degrees of freedom, with α = 0.05 used for statistical significance and I^2^ for the degree of heterogeneity. Values of I^2^ less than 25% indicated low heterogeneity, values near 50% indicated moderate heterogeneity, and those above 75% represented high heterogeneity. An I^2^ value>50% was considered indicative of substantial heterogeneity. Subgroup analyses were then conducted based on year of the study, study participants, age of participants, design, interventions, and others, and careful consideration was given to the appropriateness of the meta-analysis. If the I^2^ value was >25% or the results of an analysis clearly differed from those of other studies, a sensitivity analysis was conducted to assess the robustness of the outcomes.

Publication bias was assessed with funnel plots and Egger's test. Both random-effect and fixed-effect models were used to pool the data, and the two models yielded mainly identical results in our analysis. The results were presented from the random-effect model. All statistical analyses were performed using Stata software (Stata version 11, College Station, Texas).

## Results

**Figure 1:**
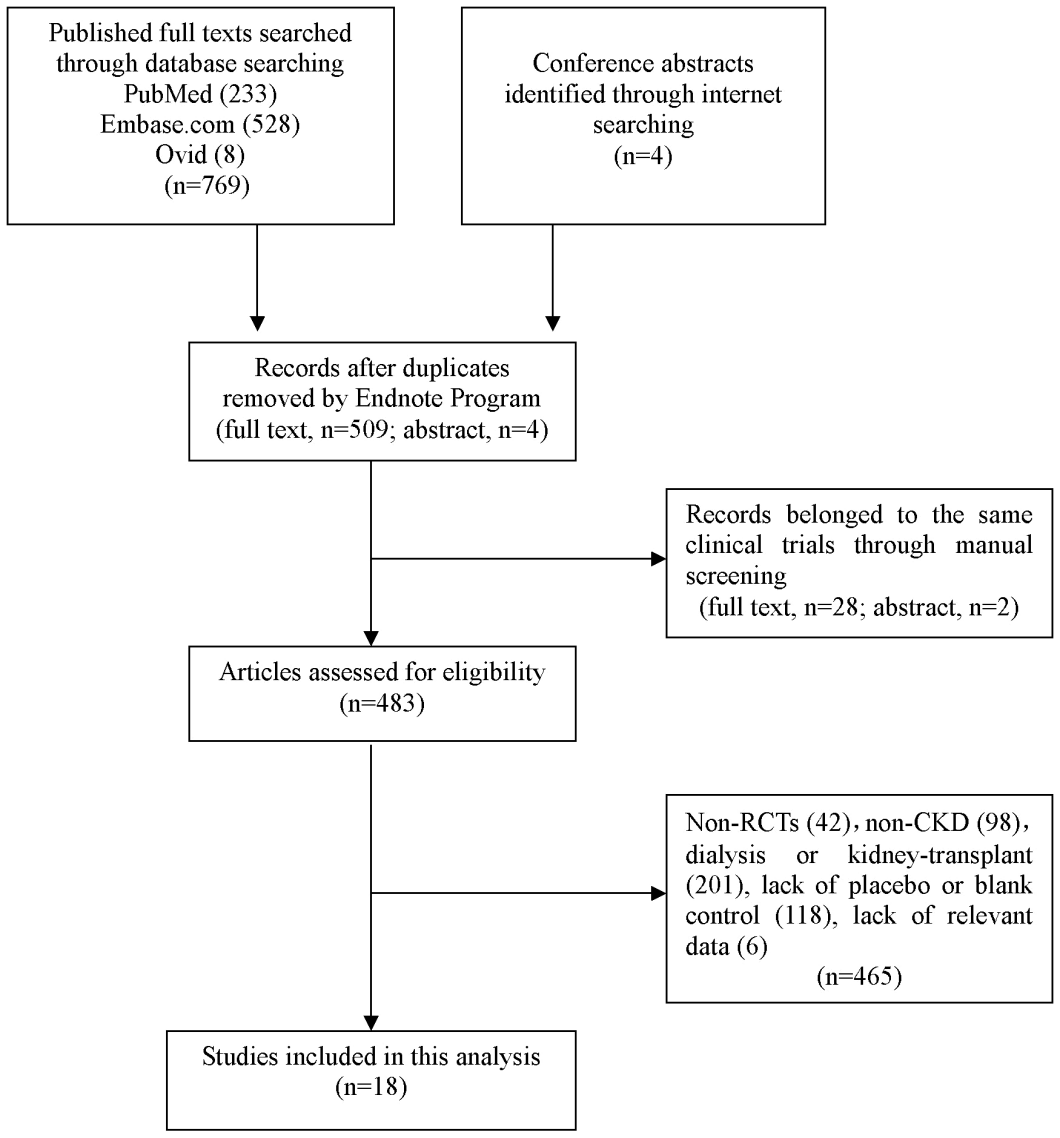
Study flow diagram for the trials selection and exclusion.

We identified 769 full-text articles via database searches and 4 abstracts via manual internet searches through September 30^th^, 2012. Of these studies, 233 were from PubMed, 528 from Embase.com, and 8 from Ovid platform. After auto screening was performed using the Endnote program, 260 duplicate articles were removed, and 509 full-text articles were identified by manual screening. Of the 4 abstracts selected, 2 were excluded for lack of relevant data, and the remaining 2 were specific for trials that were reported in full-text articles. To obtain as much accurate information as possible, we contacted five corresponding authors regarding the incomplete or vague data available in their published works. Four of these authors kindly replied, but only one provided additional information that we needed. Three full-text articles were identified from the databases we listed above but downloaded from Google Scholar. Ultimately, 18 published studies [6], [11]–[27] fulfilled our inclusion criteria. Figure 1 shows the study flow regarding trial selection and reasons for exclusion.

### Trial characteristics

A total 1836 patients between the ages of 18∼93 years, with CKD at stage 3∼5, GFR values ranging from 6∼60 ml/min/1.73 m^2^ and no apparent need for dialysis or kidney transplantation at baseline, were included. The treatment duration ranged from 1 to 24 months (median: 6 months). Six of these trials explored the effect of vitamin D on proteinuria, twelve evaluated changes in renal function, and fifteen discussed the incidence of hypercalcemia in treated subjects as compared to controls. Two other studies compared the effects of newer versus more established vitamin D compounds in non-dialysis patients (Table 2).

**Table 2:** Characteristics of the randomized controlled clinical trials involved in this analysis.

Study	Enrolled Country	Sample size	Mean age (years)	Basal disease	Renal function	Intervention Methods in study group	ACEI/ARB usage	Calcium usage	Study duration (months)	Outcomes in these trials
Nordal 1988 [11]	Norway	30	47.5	nephritis, interstitial nephritis, DM, PKD	6∼55 ml/min	Calcitriol 0.25 µg daily, then 0.5 µg daily	not informed	not informed	8	hypercalcemia
Hamdy 1995 [12]	Belgium, France, Netherland, UK	176	52.0	nephritis, HBP or DM	15∼50 ml/min	Alfacalcidol 0.25 µg daily, adjusted to 1 µg daily	not informed	When previously taken, continued	24	CCr, hypercalcemia
Coburn 2004 [13]	the USA	55	64.6	unclear	15∼59 ml/min/1.73 m^2^	Doxercalciferol 1.0 µg/d, adjusted based on iPTH	not informed	16 patients with calcium in the two groups	6	GFR
Rix 2004 [14]	Denmark	36	52.5	DM, nephritis, PKD, HBP	10∼60 ml/min	Alfacalcidol 0.25∼0.75 µg once daily	not informed	with no use of calcium	18	CCr, hypercalcemia
Agarwal 2005 [15]	USA and Poland	195	62.2	DM or other disease	15∼60 ml/min	Paricalcitol initial dose of 1∼4 µg/d	maintain concurrent therapies including ACEi/ARB	not informed	6	GFR
Coyne 2006 [16]	the USA	220	62.7	DM or other disease	15∼60 ml/min/1.73 m^2^	Paricalcitol 1 µg daily or 2 µg thrice weekly	not informed	no use of calcium	6	GFR, hypercalcemia
Alborzi 2008 [17]	the USA	24	69.5	DM, HBP, nephritis	GFR>30 ml/min	Paricalcitol 1 µg or 2 µg daily	a stable dose of an ACEi or ARB	not informed	1	GFR, proteinuria
Fishbane 2009 [18]	the USA	55	57.8	DM, HBP, nephritis, FSGS	15∼59 ml/min/1.73 m^2^	Paricalcitol 1 µg/d, adjusted based on iPTH	a stable dose of an ACEi or ARB	not informed	6	hypercalcemia proteinuria
Rucker 2009 [19]	Canada	128	69.0	DM, HBP, nephritis, PKD, obstructive nephropathy	<30 ml/min/1.73 m^2^	Vitamin D3 1000 IU/d	not informed	65 patients with calcium, comparable in the two groups	3	GFR, hypercalcemia
De Zeeuw 2010 [6]	Netherland, the USA, Denmark, Italy, Germany	281	64.3	DM	15∼59 ml/min/1.73 m^2^	Paricalcitol 1 µg/day or 2 µg/day	Stable doses of ACEi or ARB	18 patients with calcium, comparable in the two groups	6	hypercalcemia, proteinuria
Liu 2011 [20]	China	50	35.9	IgA nephropathy	>15 ml/min/1.73 m^2^	Calcitriol 0.5 µg twice weekly	RASi at least 3 months	not informed	12	GFR, proteinuria
Basturk 2011 [21]	Turkey	48	57.8	DM or other disease	CKD stage 2–4	Cholecalciferol 300,000 IU monthly	not informed	not informed	3	hypercalcemia
Alvarez 2012 [22]	the USA	46	62.5	DM, HBP	CKD stage 2–4	Cholecalciferol 50,000 IU/1∼2weeks	not informed	with no use of calcium	13	hypercalcemia
Krairittichai 2012 [23]	Thailand	91	60.7	DM	>15 ml/min/1.73 m^2^	Calcitriol 0.25 µg twice weekly, then doubled	with no use of RASi	not informed	4	GFR, hypercalcemia proteinuria
Thadhani 2012 [24]	the USA, etc. Multi- national	227	65.0	HBP, DM or other disease	15∼60 ml/min/1.73 m^2^	Paricalcitol 2 µg/d, adjusted based on serum calcium	most patients with RASi	not informed	12	GFR, hypercalcemia
Shroff 2012 [25]	UK	47	9.3 (children)	congenital abnormality, renal venous thrombosis, other disease	CKD stage 2–4	Ergocalciferol	not informed	13 children with calcium, comparable in the two groups	12	GFR, hypercalcemia
Moe 2011 [26]	the USA	47	63.6	HBP, DM or other disease	CKD stage 3–4	Doxercalcigerol 1 µg/d versus cholecalciferol 2000 IU/d	not informed	not informed	3	hypercalcemia proteinuria
Kovesdy 2012 [27]	the USA	80	68.0	DM, HBP, ischemic, hereditary	CKD stage 3–4	Paricalcitol 1∼2 µg/d versus ergocalciferol	not informed	totally 4 patients with calcium	4	hypercalcemia

GFR, glomerular filtration rate; CCr, rate of creatinine clearance; ACEi, angiotensin-converting enzyme inhibitor; ARB, angiotensin receptor blocker; RASi, rennin-angiotensin system inhibitor ; DM, diabetes mellitus; PKD, polycystic kidney disease; HBP, high blood pressure (hypertension).

### Study quality

Most trials in our analysis were of moderate quality. Random sequence generation was clearly stated in 10 of 18 trials (56%). Allocation concealment was adequate in 5 of 18 trials (28%). Blinding of participants and personnel occurred in 12 of 18 trials (67%). Blinding of the outcome assessment was reported in 12 of 18 trials (67%). By contrast, outcome data were provided incompletely in 4 of 18 trials (22%), and selective reporting was found in 3 of 18 trials (17%). The likelihood of additional sources of bias was as high as 33% for 6 trials, and these related to declarations of interests or conflicts relating to the commercial source of the funding.

### Outcome measurement

**Figure 2:**
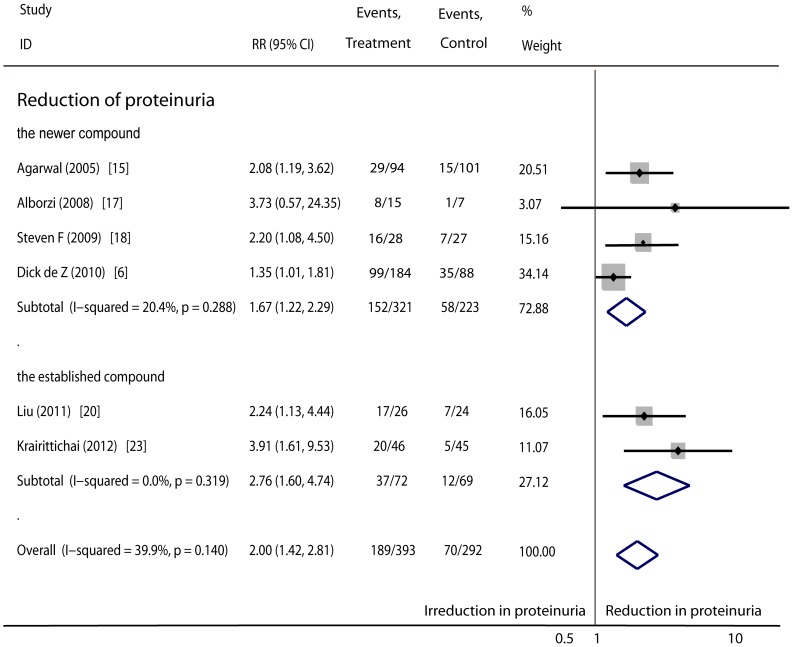
Comparison of newer and established vitamin D sterols versus controls respectively on the number of participates with reduction in proteinuria.

Proteinuria: Six RCTs (685 patients) compared the effects of vitamin D versus the use of placebo or no medication. Four of these studies evaluated a newer vitamin D analogue, and the other two evaluated an established vitamin D compound. The pooled data indicated that vitamin D reduced proteinuria in non-dialysis patients (RR, 2.00; 95%CI, 1.42 to 2.81). The RR associated with the newer vitamin D sterol was 1.67 (95%CI, 1.22 to 2.29) and that for the established compound was 2.76 (95%CI, 1.60 to 4.74) (Figure 2). The subgroup analysis showed no difference between the newer vitamin D sterol and the established one (P = 0.14). We also reviewed a study that compared the impact of the newer vitamin D analogue versus the established compound on proteinuria. To our regret, this original article did not provide concrete data, although it suggested that there was no difference between the newer compound and the established one [26].

**Figure 3:**
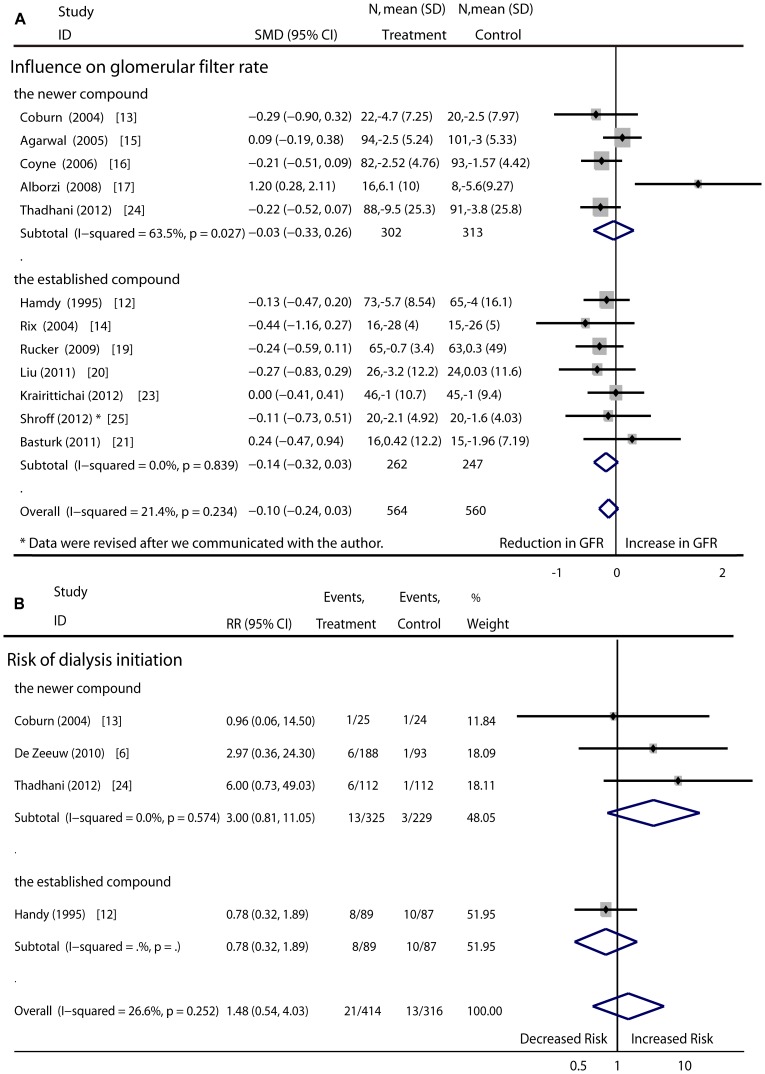
Effect of newer and established vitamin D compound on renal function versus controls respectively.

GFR: Twelve RCTs (1124 patients) evaluated the effect of vitamin D therapy on GFR. After treatment, the changes in GFR were not different (−0.10, 95%CI: −0.24 to 0.03) between the study group and the control group. Advanced analysis indicated that neither established analogues such as calcitriol and alfacalcidol (−0.14, 95%CI −0.32 to 0.03) nor newer analogues such as paricalcitol and doxercalciferol (−0.03, 95%CI −0.33 to 0.26) led to deteriorations in renal function. The subgroup analysis showed no difference between the newer vitamin D sterol and the established one (P = 0.23). No head-to-head study was obtained from the database searches that compared the effect of newer vitamin D analogues versus established compounds on GFR in non-dialysis patients (Figure 3A).

Four RCTs (730 patients) listed the numbers of patients who progressed to terminal renal failure and required dialysis. One of these trials evaluated the established vitamin D sterol, and the other three evaluated the newer compound. Neither the established compound (RR. 3.00; 95%CI, 0.81 to 11.05) nor the newer compound (RR, 0.78; 95%CI, 0.32 to 1.89) was indicated to increase the risk of renal deterioration (pooled RR, 1.48; 95%CI, 0.54 to 4.03) (Figure 3B).

**Figure 4:**
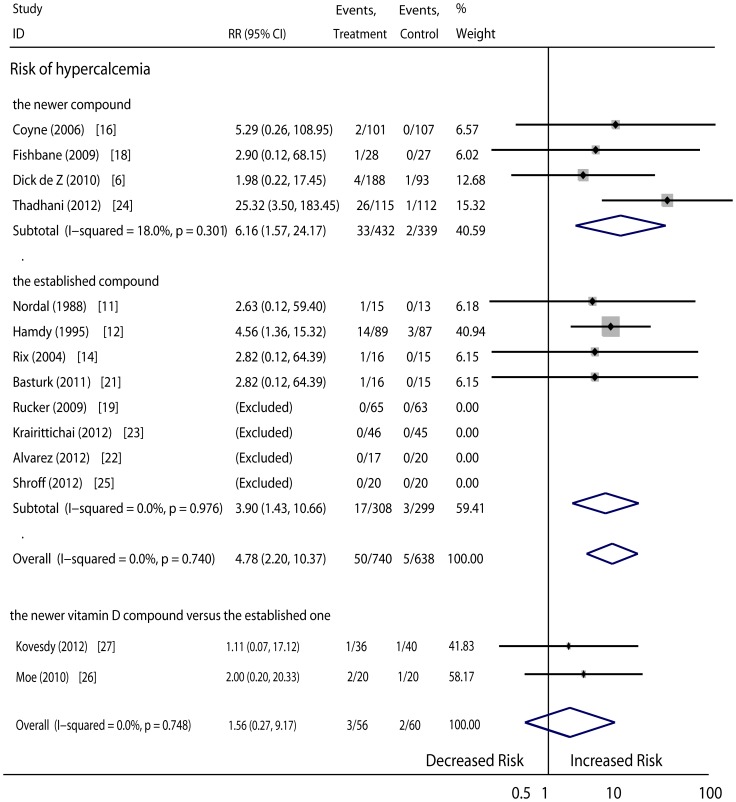
Comparison of newer vitamin D sterol and established one versus controls, and comparison of newer vitamin D versus established one on the risk of hypercalcemia.

Incidence of hypercalcemia: Regarding the occurrence of hypercalcemia, thirteen RCTs (1378 patients) compared the newer vitamin D sterol or the established compound with placebo treatment or no medication, and two RCTs compared the newer compound with the established compound. The risk of hypercalcemia was clearly higher in patients given vitamin D therapy as compared with those given the placebo or no medication (RR, 4.78; 95%CI, 2.20 to 10.37). The RR associated with the newer vitamin D compounds was 6.16 (95%CI, 1.57 to 24.17), and that associated with the established compounds was 3.90 (95%CI, 1.43 to 10.66). No difference was discovered between the newer compounds and the established compounds based on the original head-to-head studies (pooled RR, 1.56; 95%CI, 0.27 to 9.17) (Figure 4).

**Figure 5:**
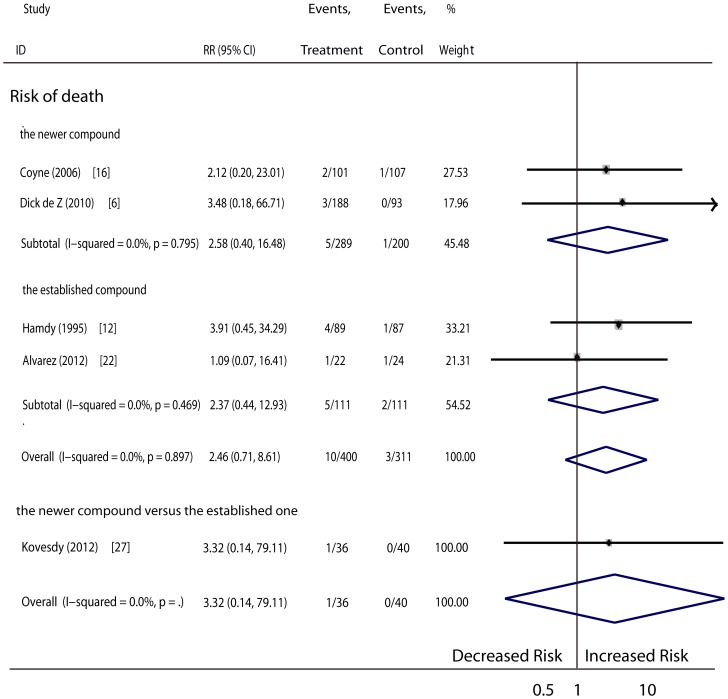
Comparison of newer and established sterols versus controls, and comparison of newer vitamin D versus established one on number of death.

**Figure 6:**
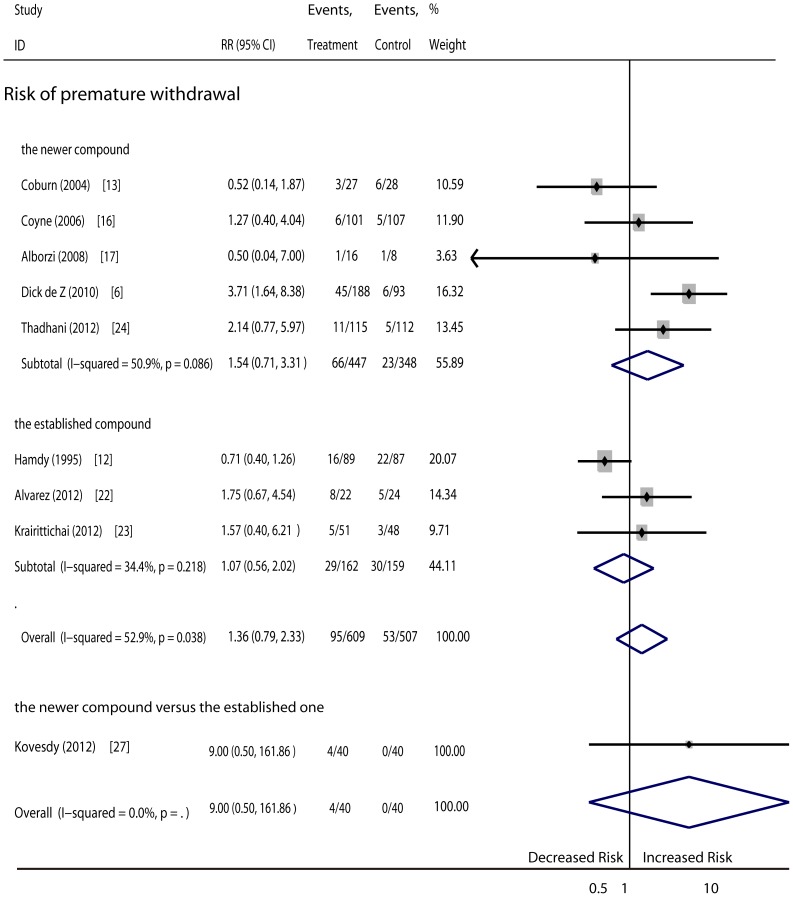
Comparison of newer and established compounds versus controls, and comparison of newer vitamin D versus established one on number of patients with premature withdrawal.

**Figure 7:**
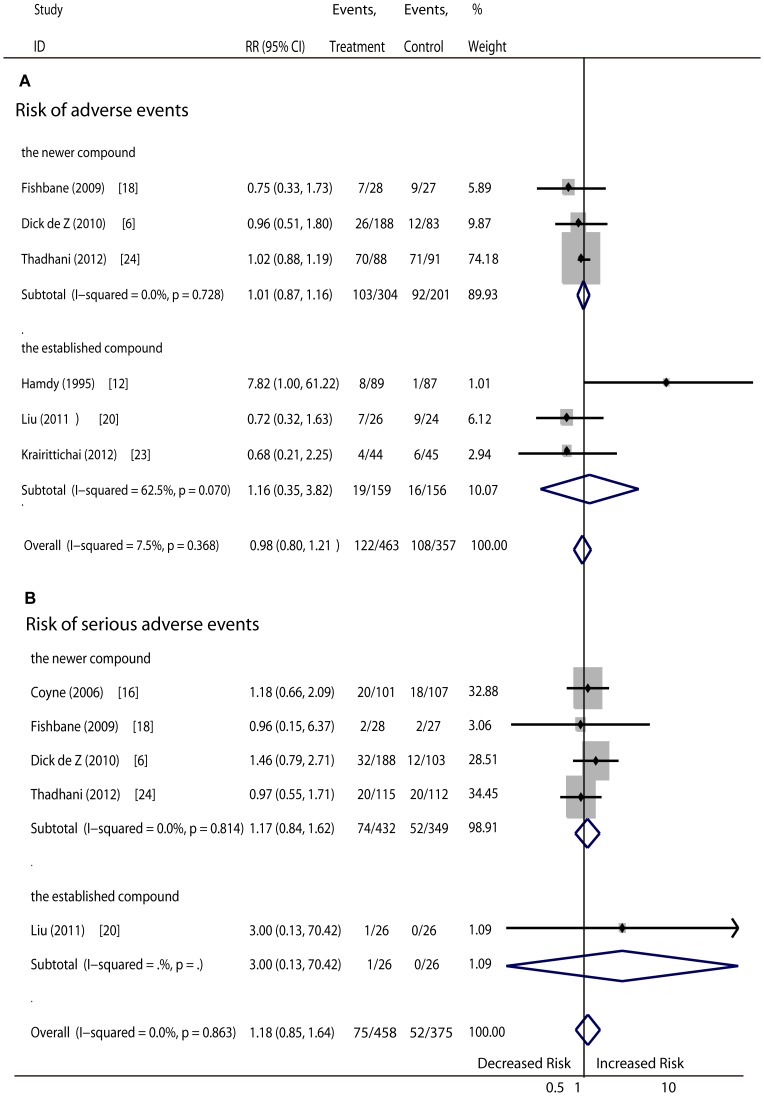
Comparison of newer and established vitamin D sterols versus controls on averse events and serious adverse events.

Other events (a total of 9 RCTs, 1221 patients): The pooled results showed no differences regarding the risk of death (Figure 5), pre-mature withdrawal (Figure 6), adverse events (Figure 7A) or serious adverse events (Figure 7B) in patients given vitamin D therapy as compare to those given the placebo or no medication. No superiority was found for either treatment with the newer vitamin D compounds or the established compounds. The reasons for patient withdrawal included serious adverse events, such as progression to dialysis or cardiac events including congestive heart failure, myocardial infarction, atrial fibrillation, acute renal failure secondary to heart failure and pericardial effusion, pneumonia, stroke, and mortality, or loss of contact. Side effects that might have been unrelated to vitamin D treatment included gastrointestinal disturbances, pseudogout, upper respiratory tract infection, cough, constipation, urinary tract infection, paronychia, diarrhea, and others. In addition, two subjects had slightly raised hepatase levels and mild anaphylaxis potentially related to vitamin D therapy (Table 3).

**Table 3:** Adverse events mentioned in the trials.

Study, year	Adverse events	Conclusions
Nordal, 1988 [11]	not informed	not informed
Hamdy, 2005 [12]	gastrointestinal disturbances, pseudogout, renal failure, dialysis, default and death	insignificant
Coburn, 2004 [13]	congestive heart failure, intestinal malabsorption, dialysis, myocardial infarction, presumed cardiac arrest, neuromuscular symptoms and other reasons	insignificant
Rix, 2004 [14]	not informed	not informed
Agarwal, 2005 [15]	not informed	not informed
Coyne, 2006 [16]	elevated liver enzyme levels, allergic reaction and death	insignificant
Alborzi, 2008 [17]	abdominal pain, acute renal failure	insignificant
Fishbane, 2009 [18]	upper respiratory tract infection, cough, constipation, abdominal cramps, headache; congestive heart failure, episode of new atrial fibrillation and pneumonia	insignificant
Rucker, 2009 [19]	not informed	not informed
De Zeeuw, 2010 [6]	diabetic gastroparesis, death, malaise, myalgia, pain, drug intolerance, erectile dysfunction, muscle spasms, edema	insignificant
Liu, 2011 [20]	upper respiratory tract infection, rash, urinary tract infection, paronychia, diarrhea, liver function disorder, hyperkalemia, joint pain, gout and renal calculus	renal calculus related to vitamin D
Basturk, 2011 [21]	not informed	not informed
Alvarez, 2012 [22]	death	insignificant
Krairittichai, 2012 [23]	upper respiratory tract infection, abnormal sweating , hyperglycemia, congestive heart failure	insignificant
Thadhani, 2012 [24]	worsening renal function and initiated long-term dialysis, other advise events	insignificant
Shroff, 2012 [25]	no ergocalciferol-related adverse events	insignificant
Moe, 2011 [26]	quality of life indices on the SF-36 questionnaire measured between treatment groups	insignificant
Kovesdy, 2012 [27]	not informed	not informed

### Heterogeneity and publication bias

Low to moderate heterogeneity was demonstrated in our analysis. The index I^2^ value from RCTs analyzing proteinuria was 39.9% (P = 0.14), that related to GFR was 21.4% (P = 0.23), and that for hypercalcemia was very low (0.0%, P = 0.74). However, the index I^2^ value from RCTs analyzing the risk for premature withdrawal was 52.9% (P = 0.04), and we explored potential reasons for this heterogeneity in the risk for premature withdrawals by subgroup analysis. We found that year of the study was a significant effect modifier and may have accounted for the heterogeneity in the premature withdrawal analysis (Table 4).

**Table 4:** Subgroup analyses to explore the reasons for heterogeneity in the trials that discussed the number of premature withdrawals.

Variable	RR (95%CI); n Trials	P value
Number of participants		0.56
≥100	1.60 (0.68 to 3.78); 4	
<100	1.16 (0.61 to 2.22); 4	
Age of participants		0.13
≤55 years	0.71 (0.40 to 1.26); 1	
55–65 years	1.62 (0.85 to 3.11); 5	
≥65 years	1.36 (0.79 to 2.34); 2	
Study duration		0.85
≥12months	1.26 (0.60 to 2.64); 3	
<12 months	1.40 (0.62 to 3.18); 5	
Type of medication		
established vitamin D sterols	1.07 (0.56 to 2.02) ; 3	0.56
newer vitamin D sterols	1.54 (0.71 to 3.31); 5	
Number of trial centers		0.56
monocenter	1.18 (0.67 to 2.08); 5	
multicenter	1.72 (0.56 to 5.28); 3	
Year of the study		0.001
before 2005	0.67 (0.40 to 1.13); 2	
2005∼2009	1.11 (0.39 to 3.14); 2	
since 2010	2.57 (1.56 to 4.23); 4	

The sensitivity analysis of trials exploring proteinuria and premature withdrawal showed a high level of robustness, and trials evaluating GFR in relation to treatment with newer vitamin D compounds showed a low level of sensitivity. The funnel plots and sensitivity analysis results can be found in Figure S1 and Figure S2, S3, S4 for detail. Publication bias was not detected for studies concerning GFR and for those evaluating hypercalcemia (for Egger's test, P = 0.45 and 0.80, respectively; Figure S1). Studies that evaluated proteinuria, mortality, premature withdrawal, and adverse effects were inadequate for the assessment of publication bias.

## Discussion

We performed a meta-analysis of available published studies to explore the effects of vitamin D therapy in non-dialysis patients and drew the conclusion that both newer vitamin D analogues and established compounds significantly reduced proteinuria in these patients. Although the clinical practice guidelines of KDIGO (Kidney Disease Improving Global Outcomes) have recommended vitamin D supplementation in patients with CKD mainly for treating mineral and bone disorders related to secondary hyperparathyroidism, recent clinical studies and experimental animal data have confirmed that the effects of vitamin D extend beyond mineral metabolism [6], [28]–[33].

Tian et al. reviewed the benefits of vitamin D therapy, which include immunomodulatory and anti-inflammatory effects, vascular effects, regulation of the RAS and certain effects on glucose metabolism [28]. In animal studies, vitamin D monotherapy obtained an equivalent effect on proteinuria as compared to ARB and double benefits when combined with ARB [5]. Furthermore, in a large cohort evaluated for the Third National Health and Nutrition Examination Survey (NHANES III), a stepwise rise in the prevalence of albuminuria was reported with vitamin D insufficiency [29]. All the above suggested a potential intrinsic anti-proteinuric property of vitamin D.

With the development of dialysis techniques and kidney transplant operations for patients with ESRD, patient lifespan has been significantly extended, but quality of patient life has declined and costs have sharply increased. Controlling proteinuria at early stages and preserving residual renal function are no doubt significant; however, it should be noted that in 1978, a study published in the Lancet magazine reported that 18 subjects with advanced CKD demonstrated deteriorated renal function after vitamin D treatment [34]. But this conclusion was questioned due to the small sample size and short study duration, and the result was not supported by subsequent trials.

In our analysis, vitamin D therapy was not found to damage renal function, although it was also clear that vitamin D failed to improve GFR. This was surprising because decreases in albuminuria were not associated with kidney function improvement. One potential reason for this disparity may have been differences between trials in terms of study subjects. Furthermore, several risk factors (with the exception of proteinuria) are shown to correlate with the deterioration of renal function. Besides, studies have also indicated that renal impairments occur in the absence of albuminuria in some patients with diabetes, despite the classic histological features of diabetic nephropathy [35]–[37]. Although proteinuria improvement and renal function protection do not occur in parallel after vitamin D therapy in our analysis, series of studies [38]–[42] have invariably inferred that albuminuria reduction is important for future renal outcomes.

The development of hypercalcemia is a potential hazard related to vitamin D therapy. Although negative results were reported in specific RCTs, the pooled results indicated an increased probability of hypercalcemia after vitamin D therapy. This result is consistent with other meta-analyses that evaluated patients at all CKD stages [43], and these findings indicate that serum calcium concentrations should be clinically monitored when CKD patients are taking vitamin D supplements.

In this analysis, we obtained no evidence of superiority for either the newer vitamin D compounds or the established compounds in terms of their impact on proteinuria, renal function, hypercalcemia or other events.

To the best of our knowledge, this is the first meta-analysis to evaluate randomized trials exploring the effects of vitamin D compounds on renal function in non-dialysis-dependent CKD patients. However, our analysis and, in some cases, the materials contributing to our analysis have limitations. Most of the trials evaluated were short-term, generally lasting no more than 2 years, which means that clinical outcomes, such as all-cause death and the occurrence of cardiovascular events, may not reflect the intrinsic effect of vitamin D therapy. Furthermore, randomized clinical trials investigating the effects of vitamin D on proteinuria were limited in number, and publication bias, although inadequate to be assessed in our analysis, may exist and could have affected the results.

In summary, vitamin D therapy appears to decrease proteinuria and have no negative influence on renal function in non-dialysis patients. Thus, this treatment appears to be safe for CKD treatment, but the occurrence of hypercalcemia should be evaluated when vitamin D is provided. Furthermore, no superiority for newer versus established vitamin D analogues is found in non-dialysis patients, which implies that other factors such as expense or availability should be the first consideration for patients and practitioners.

## Supporting Information

Figure S1 **Funnel plots with pseudo 95% confidence limits to detect potential publication bias.** The scatter plots represent individual studies for the indicated association. Egger's test for publication bias was not significant in this analysis.
(TIF)

Figure S2 **Sensitivity analysis of trials exploring the amelioration of proteinuria with vitamin D therapy showed a low level of sensitivity, which indicates a robust result.**
(TIF)

Figure S3 **Sensitivity analysis of trials evaluating GFR changes with newer vitamin D compounds therapy showed a low level of sensitivity.**
(TIF)

Figure S4 **Sensitivity analysis of trials inspecting premature withdrawal with vitamin D therapy showed a low levels of sensitivity.**
(TIF)

File S1 **Study protocol for this meta-analysis.**
(DOC)

File S2 **Database search strategies for this analysis.**
(DOC)

File S3 **PRISMA checklist of this meta-analysis.**
(DOC)
