# Supplementary figures and images for: Impact of Vitamin D on Chronic Kidney Diseases in Non-Dialysis Patients: A Meta-Analysis of Randomized Controlled Trials
Source: PLoS One. 2013 Apr 23;8(4):e61387. doi: 10.1371/journal.pone.0061387 (PMC3634086; doi:10.1371/journal.pone.0061387)

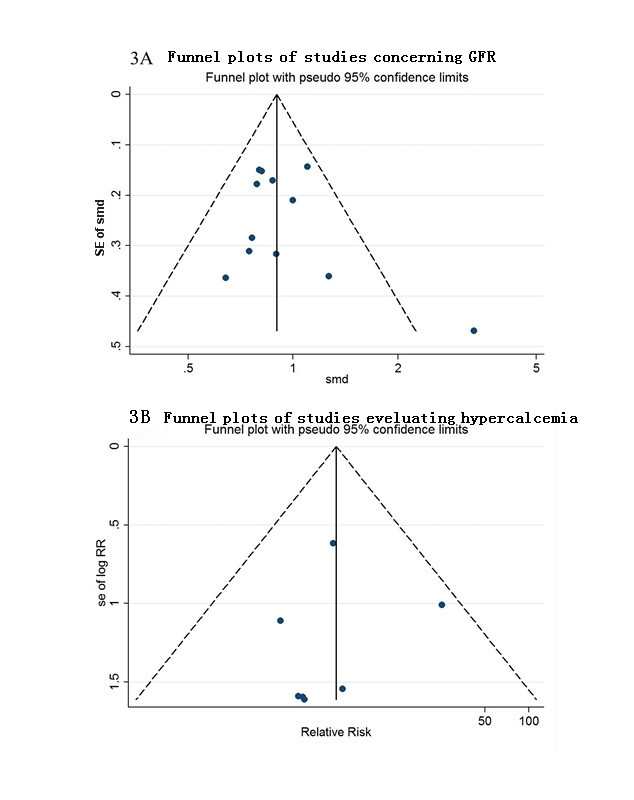

Supplement: Figure S1 — Funnel plots with pseudo 95% confidence limits to detect potential publication bias. The scatter plots represent individual studies for the indicated association. Egger's test for publication bias was not significant in this analysis. (TIF) [file pone.0061387.s001.tif]

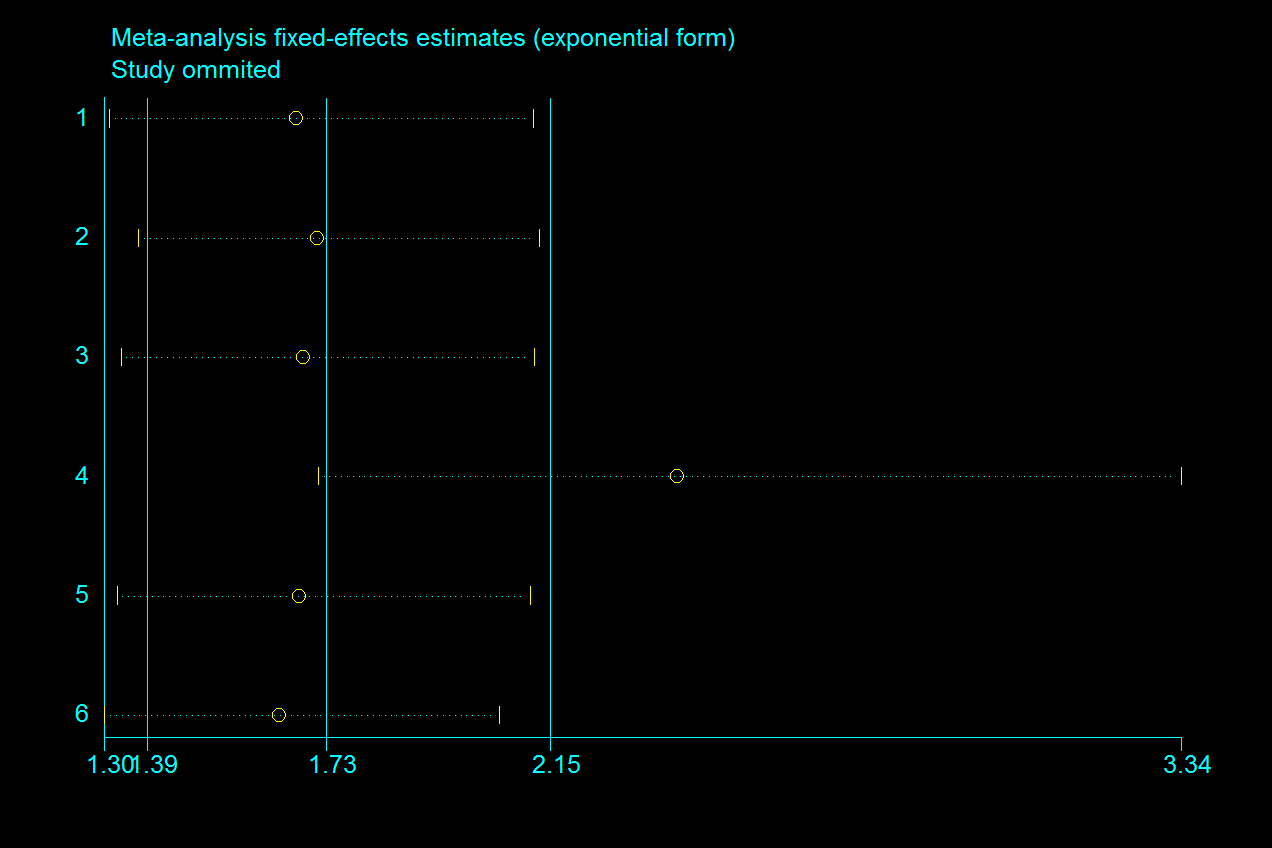

Supplement: Figure S2 — Sensitivity analysis of trials exploring the amelioration of proteinuria with vitamin D therapy showed a low level of sensitivity, which indicates a robust result. (TIF) [file pone.0061387.s002.tif]

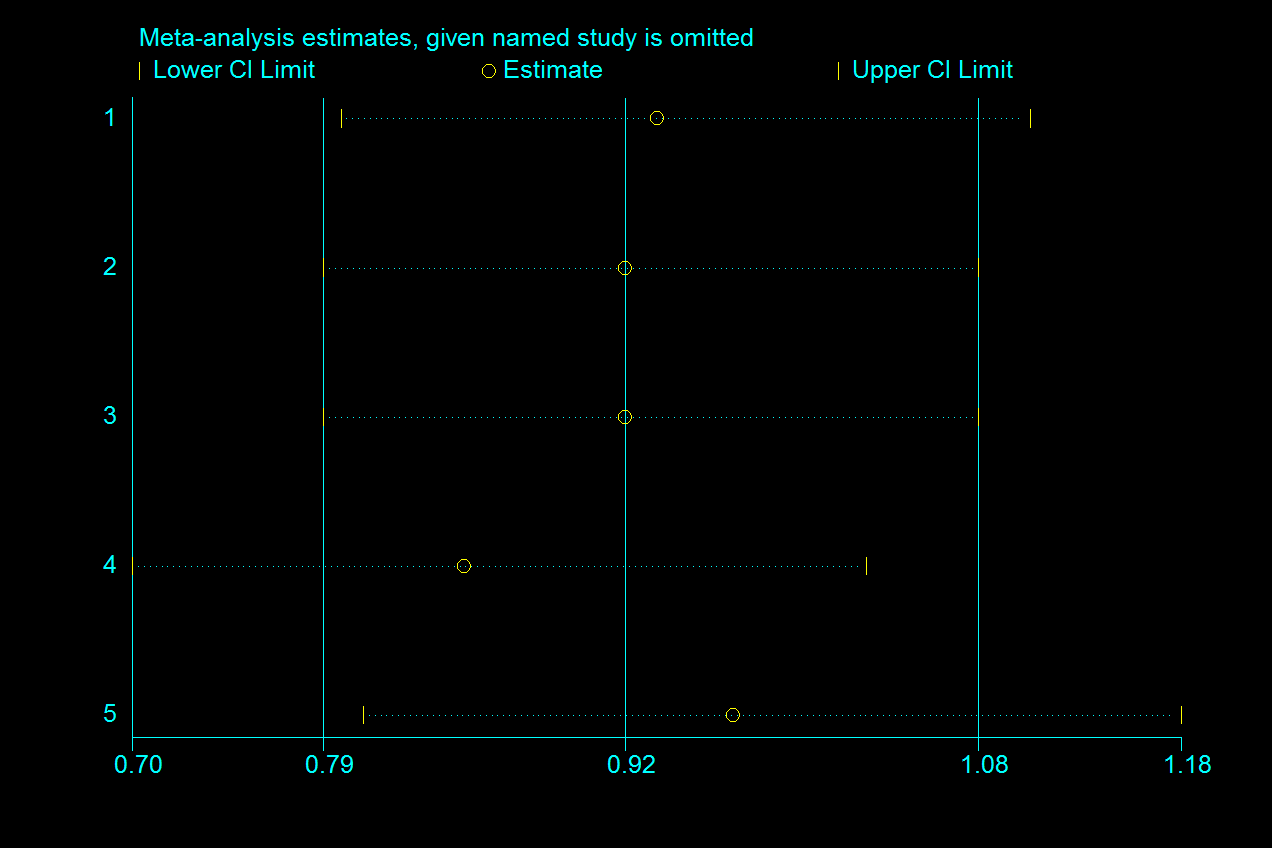

Supplement: Figure S3 — Sensitivity analysis of trials evaluating GFR changes with newer vitamin D compounds therapy showed a low level of sensitivity. (TIF) [file pone.0061387.s003.tif]

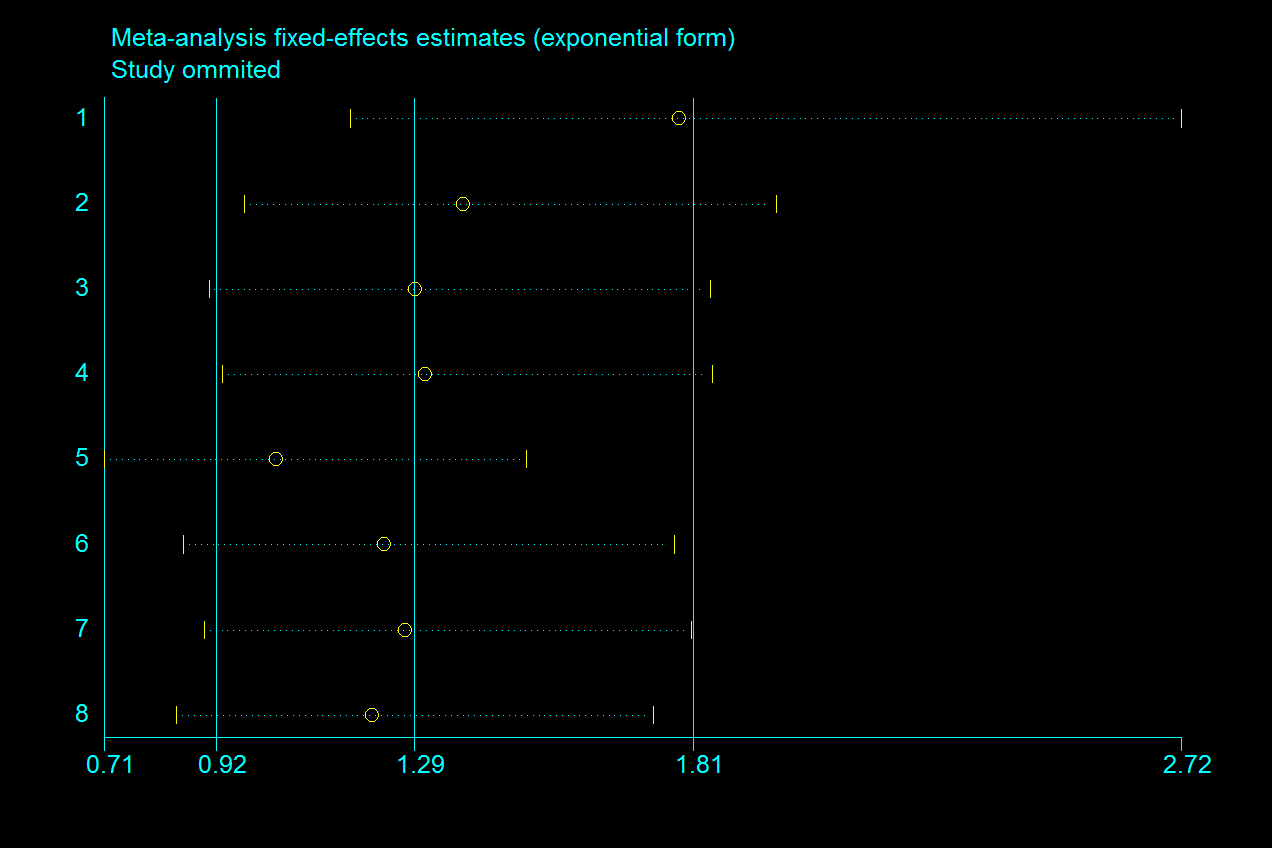

Supplement: Figure S4 — Sensitivity analysis of trials inspecting premature withdrawal with vitamin D therapy showed a low levels of sensitivity. (TIF) [file pone.0061387.s004.tif]
