# Supplementary material for: Impact of Vitamin D on Chronic Kidney Diseases in Non-Dialysis Patients: A Meta-Analysis of Randomized Controlled Trials
Source: PLoS One. 2013 Apr 23;8(4):e61387. doi: 10.1371/journal.pone.0061387 (PMC3634086; doi:10.1371/journal.pone.0061387)
Supplement: File S1 — Study protocol for this meta-analysis. (DOC) [file pone.0061387.s005.doc]

**Impact of vitamin D on chronic kidney diseases in non-dialysis patients : a Meta-analysis protocol of Randomized Controlled Trials**

Lijuan Xu, Fangfang Zeng and Yanbing Li

**Abstract**

**Introduction:** Recent studies have supported a role for both newer and established vitamin D compounds in improving proteinuria but lack of systematical evaluation. Concerns remain regarding the influence of vitamin D on the progression of renal function in non-dialysis patients.

**Methods and analysis:** We will analyze the efficacy and safety of vitamin D in non-dialysis patients and compare the differences of the newer versus the established vitamin D compound by performing a meta-analysis of randomized controlled trials. We assess that randomized controlled clinical trials (RCTs) with patients receiving vitamin D in the study group and patients receiving placebo or no medications as controls are eligible for our analysis. RCTs that comparing newer vitamin D compound with established one will also be enrolled in our analysis. Subjects in the original trials should have no need for dialysis or renal transplantation at baseline. We will consider the changes of albuminuria, glomerular filter rate, the risk of renal deterioration, hypercalcemia and other adverse effects in these trials. Those with relevant incomplete parameters required for our analysis as published and unobtainable from the authors of the respective studies will be excluded. Two authors will examine the full text of all remaining papers to determine eligibility, extract data and assess risk of bias independently. Where studies are sufficiently homogeneous, the results will be synthesized using a fixed-effect model. Otherwise, a random-effect model will be utilized. Standardized mean differences will be presented for continuous outcomes and relative ratios will be used for binary outcomes.

**Dissemination:** The meta analysis is hope to be published in a journal and be disseminated electronically or in print. Brief reports of the outcomes will be disseminated to appropriate audiences via conference communication.

**Introduction**

End-stage renal disease (ESRD) imposes heavy health and economic burdens on the individuals. Risk factors affecting kidney diseases include baseline urinary albumin excretion, glycemic control, age and blood pressure. Microalbuminuria is one the earliest clinical manifestations of nephropathy and is associated with substantial risk for progressive kidney disease. Additionally, albuminuria predicts cardiovascular events, all-cause mortality and hospitalization for congestive heart failure. Recent data have shown that increase of proteinuria and reduction of glomerular filtration rate (GFR) are independent predictors of all-cause mortality. So reduction of proteinuria and protection of kidney function at stages before dialysis are pivotal for preventing long-term kidney loss and other adverse events. It is urgently required to explore therapeutic modalities for CKD treatment.

Animal experiments have revealed that vitamin D can reduce proteinuria. But the majority of existing clinical data focused on the effect of vitamin D on mineral metabolism and bone diseases of secondary hyperparathyroidism. Among the limited clinical studies exploring other aspects of vitamin D, VITAL trial, one of the well designed, relatively large-scale studies showed an inspiring but borderline significant results of albuminuria improvement. In addition, whether vitamin D has potential harms to renal function is also in suspense. Widely used in patients with CKD, vitamin D therapy has been historically based on ergocalciferol (vitamin D2), cholecalciferol (vitamin D3), calcitriol (1, 25 dihydroxyvitamin D3) and alfacalcidol ( 1α- hydroxyvitamin D3). But the newer vitamin D analogues, i.e. paricalcitol, doxercalciferol, oxacalcitriol and falecalcitriol play a more and more important role in CKD treatment. As far as we know, limited comprehensive meta-analysis and systematic reviews have explored the influence of vitamin D on proteinuria and progression of CKD in non-dialysis patients, or compare the difference between the newer sterols and the established ones. So we will perform a meta-analysis to clarify these issues. Hypercalcemia and other adverse events will be further evaluated.

**Objectives**

1. To determine the efficacy of vitamin D compounds on proteinuria, the progression of renal function in non-dialysis patients with chronic kidney diseases.
2. To evaluate the safety of vitamin D compounds on the risk of hypercalcemia and other events in non-dialysis patients.
3. To compare the difference between the newer vitamin D compounds and the established ones in non-dialysis patients.

**Methods and analysis**

**Database selection**

We choose PubMed, EMBASE.com and the OvidSP as our searching sources. Google Scholar will also be searched as an additional measure for full-text articles. PubMed is chosen because of its more-than-22-million citations from 1975 for biomedical literature from Medline, life science journals, and online books. It is a free resource and convenient to be searched in at any place and any time. Embase.com is a highly versatile database covering the most important international biomedical literature from 1966 to the present day. All Medline records produced by the National Library of Medicine (NLM) are included in that searching engine. OVID EBM Reviewer is an important database obtaining information of evidence-based medicine. It comprises seven sub-databases of evidence based medicine including the Cochrane library. The databases selected cover comprehensive information for published studies.

Abstracts presented at the meetings of the American Society of Nephrology, National Kidney Foundation and World Congress of Nephrology, American Diabetes Association, European Association for the Study of Diabetes and International Diabetes Federation in recent years will be searched for additional studies.

**Participants**

Participants may include those of all ages who suffered chronic kidney diseases at baseline, and with therapy of newer or established vitamin D compounds. No restriction is set for the causes of chronic kidney diseases, age, profession or gender. Those with apparent need for dialysis or renal transplantation at baseline will be excluded.

**Interventions**

Interventions will be included all types of vitamin D compounds supplementation irrespective of dosage and dosage form. The established vitamin D compounds include ergocalciferol (vitamin D2), cholecalciferol (vitamin D3), calcitriol (1, 25 dihydroxyvitamin D3) and alfacalcidol ( 1α- hydroxyvitamin D3). While the newer vitamin D analogues are in the form of paricalcitol, doxercalciferol, oxacalcitriol and falecalcitriol.

**Comparisons**

Comparisons will include no intervention or placebo controls. Comparison of the newer versus the established vitamin D compounds will also be involved.

**Study design**

Studies with the design of randomized controlled clinical trials will be included. There will be no criteria for year of study publication, country, language or the length of follow-up.

**Primary outcomes**

1. Efficacy of vitamin D compounds on the decrease of proteinuria compared with placebo or blank control. Proteinuria is assessed either by urine albumin/ creatinine or by 24-hour protein excretion.
2. Efficacy of vitamin D compounds on the progression of renal function which is evaluated compared with placebo or blank control by calculating glomerular filtration rate or creatinine clearance rate.
3. Risk of vitamin D compounds on hypercalcemia compared with placebo or blank control.
4. The difference of these parameters between the newer vitamin D compound and the established one.

**Secondary outcomes**

1. Risk of death, premature withdrawal, adverse events and serious adverse events with vitamin D therapy in these studies.
2. The difference of these parameters between newer and established vitamin D compound in these studies.

**Search strategies**

PubMed:

#1 vitamin d;

#2 vitamin d2;

#3 vitamin d3;

#4 calciferol;

#5 alfacalcidol;

#6 calcitriol;

#7 kidney disease;

#8 nephropathy;

#9 renal failure;

#10 #1 or #2 or #3 or #4 or #5 or #6;

#11 #7 or #8;

#12 #10 and #11 with the limitation of clinical trials.

EMBASE:

1 vitamin d;

2 vitamin d2;

3 vitamin d3;

4 calciferol;

5 alfacalcidol;

6 calcitriol;

7 or/ 1-6;

8 kidney disease;

9 nephropathy;

10 renal failure;

11 or/ 8-10;

12 and/ 7, 11;

13 clinical trial;

14 and/ 12, 13

OvidSP:

1 vitamin d;

2 vitamin d2;

3 vitamin d3;

4 calciferol;

5 alfacalcidol;

6 calcitriol;

7 kidney disease;

8 nephropathy;

9 renal failure;

10 1 or 2 or 3 or 4 or 5 or 6;

11 7 or 8;

12 9 and 10.

**Data extraction**

The following information will be summarized in a predefined data collection form: title, the first author’s name, nation, year of publication, controls and mean age. For binary outcomes, number of cases and controls will be recorded. For continuous data, numbers, mean values and standard deviations of changes from baseline in the study group and the control group will be recorded, respectively. Standard deviation will be calculated based on the equation advised in Cochrane Handbook if 95% confidence interval is provided rather than standard deviation. If the baseline and final standard deviations are given, while the changes of standard deviations are unknown, the correlation coefficient method advised by Follmann will be used to calculate the values. Where studies are sufficiently homogeneous, the results will be synthesized by using a fixed-effect model. Otherwise, a random-effect model will be utilized.

Two reviewers will screen the search results based on the inclusion and exclusion criteria. Studies that do not meet the criteria will be excluded at this stage. The two reviewers will independently extract useful data from the selected trials. When it is considered desirable and potentially useful, we will contact the investigators for additional information. Discrepancies in data extraction and quality evaluation between the two reviewers will be arbitrated by an arbitrator. Relevant missing data will be sought by contacting the original author of the respect study.

**Risk of bias assessment**

Two reviewers will independently assess the risk of bias in eligible studies by assessing the adequacy of the characteristics described in the Cochrane Handbook: adequate sequence generation, allocation concealment, blinding, incomplete outcome data addressed, selective reporting, and other bias. Disagreement will be resolved through discussion between the two reviewers.

**Assessment of study heterogeneity and sensitivity**

Heterogeneity between studies will be assessed using the Istatistic. An Ivalue >50% will be considered indicative of substantial heterogeneity, subgroup analyses will be conducted based on participants, design, interventions, outcomes and study quality and careful consideration will be given to the appropriateness of meta-analysis. If an I value is higher than 25% or the result is obviously different, a sensitivity analysis will be conducted to assess the robustness of the outcomes.

**Assessment of reporting bias**

Funnel plots of eligible studies and Egger’s test will be used to assess bias that might arise through selective reportings.

**Discussion**

This analysis will provide evidence of the efficacy and safety of different vitamin D compounds on proteinuria and the progression in non-dialysis patients. It will be of benefit to researchers and policy makers in management chronic kidney diseases at stages prior to dialysis.

**References**

1. Collins AJ, Foley RN, Chavers B, et al. United States Renal Data System 2011 Annual Data Report. Am J Kidney Dis 59: e1-420, 2012.
2. De Zeeuw D, Remuzzi G, Parving HH, et al. Albuminuria, a therapeutic target for cardiovascular protection in type 2 diabetic patients with nephropathy. Circulation 110: 921-927, 2004.
3. Knobler H, Zornitzki T, Vered S, et al. Reduced glomerular filtration rate in asymptomatic diabetic patients: predictor of increased risk for cardiac events independent of albuminuria. J Am Coll Cardiol 44: 2142-2148, 2004.
4. Salles GF, Cardoso CR, Pereira VS, Fiszman R, Muxfeldt ES. Prognostic significance of a reduced glomerular filtration rate and interaction with microalbuminuria in resistant hypertension: a cohort study. J Hypertens 29: 2014-2023, 2011.
5. Tobe SW, Clase CM, Gao P, et al. Cardiovascular and renal outcomes with telmisartan, ramipril, or both in people at high renal risk: results from the ONTARGET and TRANSCEND studies. Circulation 123: 1098-1107, 2011.
6. Chan KE, Ikizler TA, Gamboa JL, Yu C, Hakim RM, Brown NJ. Combined angiotensin-converting enzyme inhibition and receptor blockade associate with increased risk of cardiovascular death in hemodialysis patients. Kidney Int 80: 978-985, 2011.
7. Titan SM, M Vieira J Jr, Dominguez WV, Barros RT, Zatz R. ACEI and ARB combination therapy in patients with macroalbuminuric diabetic nephropathy and low socioeconomic level: a double-blind randomized clinical trial. Clin Nephrol 76: 273-283, 2011.
8. Zhang Y, Deb DK, Kong J, et al. Long-term therapeutic effect of vitamin D analogue doxercalciferol on diabetic nephropathy: strong synergism with AT1 receptor antagonist. Am J Physiol Renal Physiol 297: F791-801, 2009.
9. Uhlig K, Berns JS, Kestenbaum B, et al. KDOQI US commentary on the 2009 KDIGO Clinical Practice Guideline for the Diagnosis, Evaluation, and Treatment of CKD-Mineral and Bone Disorder (CKD-MBD). Am J Kidney Dis 55: 773-799, 2010.
10. De Zeeuw D, Agarwal R, Amdahl M, et al. Selective vitamin D receptor activation with paricalcitol for reduction of albuminuria in patients with type 2 diabetes (VITAL study): a randomised controlled trial. Lancet 376: 1543-1551, 2010.
11. Dark P, Wilson C, Blackwood B, Danny F McAuley, Gavin D Perkins, Ronan McMullan et al. Accuracy of LightCycler SeptiFast for the detection and identification of pathogens in the blood of patients with suspected sepsis: a systematic review protocol. BMJ Open 2:e000392, 2012.
12. Effiong A, Effiong AI. Palliative care for the management of chronic illness: a systematic review study protocol. BMJ Open 2:e000899, 2012.
13. Follmann D, Elliott P, Suh I, Cutler J. Variance imputation for overviews of clinical trials with continuous response. J Clin Epidemiol 45(7):769-773, 1992.
