# Supplementary material for: Impact of Vitamin D on Chronic Kidney Diseases in Non-Dialysis Patients: A Meta-Analysis of Randomized Controlled Trials
Source: PLoS One. 2013 Apr 23;8(4):e61387. doi: 10.1371/journal.pone.0061387 (PMC3634086; doi:10.1371/journal.pone.0061387)
Supplement: File S2 — Database search strategies for this analysis. (DOC) [file pone.0061387.s006.doc]

**Database search strategies**

**PubMed:**

#1 vitamin d;

#2 vitamin d2;

#3 vitamin d3;

#4 calciferol;

#5 alfacalcidol;

#6 calcitriol;

#7 kidney disease;

#8 nephropathy;

#9 renal failure;

#10 #1 or #2 or #3 or #4 or #5 or #6;

#11 #7 or #8;

#12 #10 and #11 with the limitation of clinical trials.

**EMBASE.com:**

1 vitamin d;

2 vitamin d2;

3 vitamin d3;

4 calciferol;

5 alfacalcidol;

6 calcitriol;

7 or/ 1-6;

8 kidney disease;

9 nephropathy;

10 renal failure;

11 or/ 8-10;

12 and/ 7, 11;

13 clinical trial;

14 and/ 12, 13

**OvidSP:**

1 vitamin d;

2 vitamin d2;

3 vitamin d3;

4 calciferol;

5 alfacalcidol;

6 calcitriol;

7 kidney disease;

8 nephropathy;

9 renal failure;

10 1 or 2 or 3 or 4 or 5 or 6;

11 7 or 8;

12 9 and 10.
